# The Synthesis of 1-(4-Triethoxysilyl)phenyl)-4,4,4-trifluoro-1,3-butanedione, a Novel Trialkoxysilane Monomer for the Preparation of Functionalized Sol-gel Matrix Materials

**DOI:** 10.3390/molecules13102601

**Published:** 2008-10-20

**Authors:** Christopher J. Peeples, Raghu Ram Earni, John C. DiCesare

**Affiliations:** 800 South Tucker Drive, Department of Chemistry and Biochemistry, The University of Tulsa, Tulsa, Oklahoma, 74104, USA

**Keywords:** Trialkoxysilane, 4,4,4-Trifluoro-1-phenyl-1,3-butanedione (4-TPB), Sol-gel, Molecular imprinting

## Abstract

The title compound, 1-(4-triethoxysilyl)phenyl)-4,4,4-trifluoro-1,3-butanedione, was synthesized in a three-step sequence starting from 2-(4-bromophenyl)propene. Containing both a trialkoxysilyl and a substituted 1,3-butanedione functional grouping within its structure, this new silane is a viable starting material for the preparation of functionalized sol-gel materials.

## Introduction

The use of molecular imprinting on a silica sol-gel thin film constitutes an important process for the development of chemical sensor materials [1,2,3,4,5,6]. In our present efforts to develop a fluorometric sensing material for organophosphonate nerve agents using a templated sol-gel matrix incorporating the Eu(III) cation [7,8,9], initial studies using the ligand 4,4,4-trifluoro-1-phenyl-1,3-butanedione (**1**) seemed to indicate that the 4,4,4-trifluoro-1,3-butanedionyl moiety was the superior binding group for use in forming the desired ligand-Eu(III) complex. In view of this observation, it was envisioned that the use of an appropriate trialkoxysilyl monomer incorporating this binding group such as the silane 1-(4-(triethoxysilyl)phenyl)-4,4,4-trifluoro-1,3-butanedione (**2**) would allow for the covalent integration of the ligand-Eu(III) complex into the sol-gel matrix in the initial stages of the overall molecular-imprinting process. In this study, we wish to report the successful preparation of **2**, a new triethoxysilane monomer that we are using in this study.

**Figure 1:**
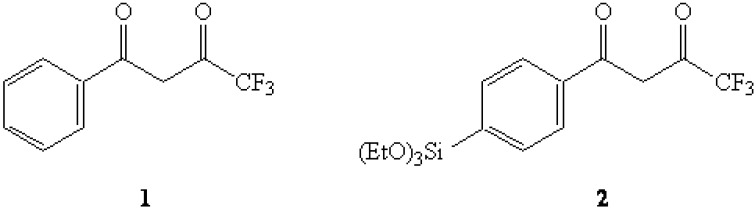
4,4,4-Trifluoro-1-phenyl-1,3-butanedione (**1**) and the title compound **2**.

## Results and Discussion

In our attempts to prepare **2**, the two main issues of interest were the successful introduction of both the triethoxysilyl and 1,3-diketone functionalities and the use of reaction conditions that would not hydrolyze the acid-labile triethoxysilyl portion of the molecule. While this sensitivity to acid hydrolysis led to some difficulties initially, we were eventually successful in devising an efficient and cost-effective synthesis for this potentially-useful and interesting silane monomer.

In the first of our attempts, the dimethyl ketal of 4-bromoacetophenone was converted to the corresponding Grignard reagent which was then reacted with tetraethoxysilane (TEOS) to afford the expected triethoxysilyl dimethyl ketal **3**. It was thought that a selective hydrolysis of the ketal grouping might yield the anticipated ketone 4-(triethoxysilyl)acetophenone (**7**) which could then be acylated *via* a Claisen condensation to afford **2**. However, numerous difficulties were encountered in our attempts to selectively-hydrolyze the ketal with the triethoxysilyl group also being so affected.

**Figure 2:**
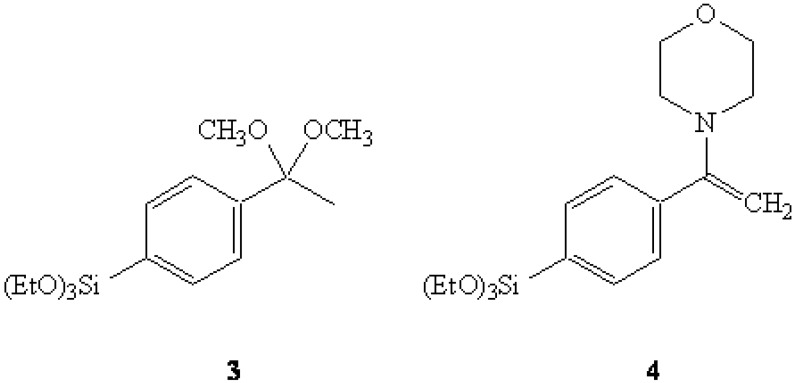
Ketal **3** and Enamine **4**.

In another related attempt, the morpholine enamine derived from 4-bromoacetophenone was submitted to the same Grignard sequence to yield the anticipated triethoxysilyl enamine **4**. It was expected that the acylation of 4 with trifluoroacetic anhydride [10], followed up with the "traditional" Stork work-up, could potentially afford **2**. However, the same difficulties with over-hydrolysis were encountered here as well. A successful synthesis of **2** was accomplished by employing a more traditional approach using 2-(4-bromophenyl)propene (**5**) as the primary starting material [11,12]. The Grignard reagent of **5** was reacted with TEOS to give 2-(4-(triethoxysilyl)phenyl)propene (**6**) in 81% yield.

**Scheme 1:**
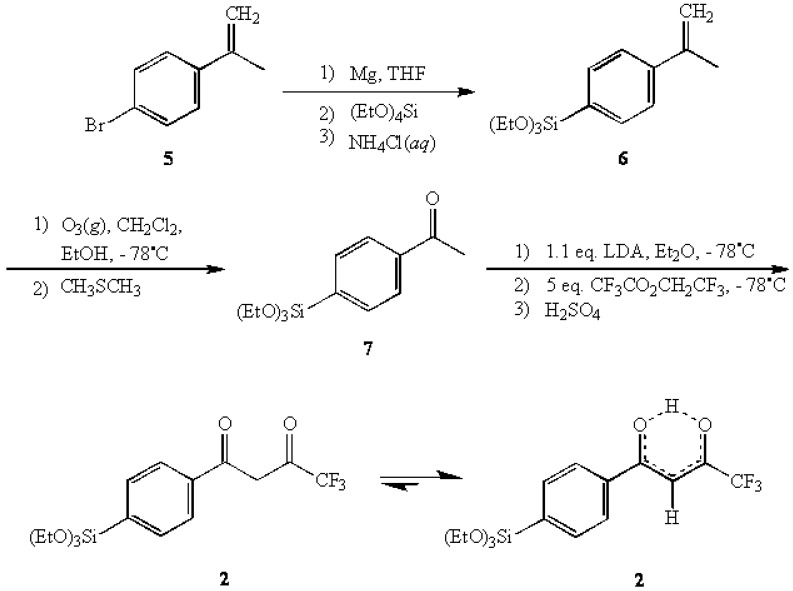
The synthesis of **2** form 2-(4-bromopheny)propene (**5**).

The subsequent ozonolysis of **6** afforded 4-(triethoxysilyl)acetophenone (**7**) [13,14] in 70% yield which was then reacted with a five-fold excess of 2,2,2-trifluoroethyl trifluoroacetate in an LDA-directed Claisen condensation [15] to provide the title compound 1-(4-(triethoxysilyl)-phenyl)-4,4,4-trifluoro-1,3-butanedione (**2**) in 73% yield. In characterizing **2**, ^1^H-NMR analysis indicated that the enol tautomer was the predominant form present (>98%) with virtually none of the keto form being detected.

## Conclusions

The route as devised for the preparation of **2** represents an efficient and cost-effective process that is readily amendable to scale-up. Following our successful synthesis of **2**, subsequent follow-up studies have indicated that this monomer complexes quite well with the Eu(III) ion and does exhibit fluorometric activity in the presence of our VX-simulant compounds making it a viable candidate for fabricating our desired sol-gel materials. This encouraging observation tends to imply that this monomer may have other potential uses within the realm of molecular imprinting and templated sol-gel production. The results of our current efforts in this project will be the focus of future journal articles.

## Experimental

### General

All reactions were run under dry N_2_. Any reactions involving the generation or use of either Grignard reagents or LDA were run in glassware previously flame-dried under vacuum. Anhydrous acetone, TEOS, dimethylsulfide, anhydrous diethyl ether, and 2,2,2-trifluoroethyl trifluoroacetate were obtained from Aldrich and were used as received. LDA was obtained in the form of a commercial solution from Aldrich (1.5 M solution in cyclohexane) and was used as received. Anhydrous THF was prepared by distillation from a solvent still containing Na/benzophenone ketal immediately prior to use. Vacuum distillations were carried out using a Kugelrohr apparatus with all boiling points being reported at 0.20 mmHg. A PCI ozonizer (1 lb/day generation capacity) was used for the ozonolysis reactions with dry O_2_ being used as the feed gas source. FT-IR spectra were obtained using a Nicolet Avatar 360 spectrophotometer with NaCl plates being used for all thin films. ^1^H-NMR and ^13^C-NMR spectra were obtained using a Varian Unity Plus 300 MHz spectrometer and were measured at 300 and 75 MHz respectively, with all chemical shifts reported in ppm using TMS as the internal standard and all determined J values reported in Hz.

*2-(4-(Triethoxysilyl)phenyl)propene* (**6**). Into a 250-mL round-bottom three-neck flask were placed magnesium turnings (875 mg, 36.0 mmol) and a magnetic stirring bar and the resulting assembly was flame-dried under vacuum. The flask was then fitted with a reflux condensor and pressure-equalized dropping funnel, was flame-dried under a stream of dry N_2_ and allowed to cool to RT. The dropping funnel was charged with a solution of 2-(4-bromophenyl)propene (**5**) (5.91 g, 30.0 mmol) in anhydrous THF (60 mL). Approximately 15 mL of the bromide-THF solution was discharged from the dropping funnel, 1,2-dibromoethane (four drops) was added to the solution in the flask and the resulting mixture was heated to reflux and stirred until the formation of the Grignard reagent had commenced (*circa* 10 min). The remainder of the bromide-THF solution was then added dropwise with continued heating and stirring over the course of 1 h and the reaction mixture was refluxed with stirring for an additional 90 min. The reaction mixture was allowed to cool to RT, a solution of tetraethoxysilane (6.87 g, 7.35 mL, 33.0 mmol) in anhydrous THF (10 mL) was added rapidly with stirring over the course of 2 min, and the resulting mixture was refluxed with continued stirring for an additional 16 h. The reaction mixture was then allowed to cool to RT, was cooled to 0-5^°^C by immersion in an icebath, sat. NH_4_Cl(*aq*) (15 mL) was added dropwise with vigorous stirring over the course of 5 min and the resulting mixture was stirred for an additional 10 min with continued cooling. The mixture was filtered through Celite^®^ and the collected magnesium salts were washed with three portions of ether (50 mL each). The combined organic extracts were washed with water and saturated aqueous NaCl, dried (MgSO_4_) and concentrated *in vacuo* to afford the crude silane product as a thick, golden-yellow oil that was purified by vacuum distillation: 6.78 g (24.2 mmol, 81%); isolated as a clear, colorless thick oil; bp 92-95^°^C (Kugelrohr, 0.20 mmHg); FT IR (thin film) 1628 (mod.), 1616 (w) cm^-1^; ^1^H-NMR (CDCl_3_) δ 7.69 (2H, d, *J* = 8.1 Hz), 7.52 (2H, d, *J* = 8.1 Hz), 5.45 (1H, d, *J*_geminal_ = 1.5 Hz), 5.15 (1H, d, *J*_geminal_ = 1.5 Hz), 3.92 (6H, q, J = 6.3 Hz), 2.19 (3H, s), 1.29 (9H, t, J = 6.3 Hz); ^13^C- NMR (CDCl_3_) δ 143.4, 143.3, 135.1, 130.1, 125.2, 113.3, 59.0, 21.9, 18.50.

*4-(Triethoxysilyl)acetophenone* (**7**). Into a 200-mL round-bottom three-neck reaction flask equipped with a magnetic stirring bar, a fire-polished bubbler tube, a glass stopper and a gas outlet portal was placed a solution of **6** (3.00 g, 10.7 mmol) dissolved in a 1:4 (v/v) mixture of dry ethanol in dichloromethane (60 mL) and the stirred solution was cooled to -78^°^C. With continued cooling and stirring, the solution was treated with O_3_ for 5 min [16]. Following the purgement of excess ozone gas from the solution, dimethyl sulfide (3.5 mL, 3.0 g, 48.3 mmol) was added to the cooled and stirred solution over the course of 2 min and the resulting mixture was allowed to warm to and stir at RT for an additional 12 h. The reaction mixture was concentrated *in vacuo* and the isolated residue was dissolved into ether (50 mL). The resulting ethereal solution was washed with two portions of water (50 mL each) and saturated aqueous NaCl, dried (MgSO_4_) and concentrated *in vacuo* to afford the crude ketone product as a thick, light-yellow oil that was purified by vacuum distillation: 2.12 g (7.51 mmol, 70%); isolated as a clear, colorless thick oil; bp 128-131^°^C (Kugelrohr, 0.20 mmHg); FT IR (thin film) 1689 cm^-1^ (vs, C=O); ^1^H-NMR (CDCl_3_) δ 7.95 (2H, d, *J* = 8.1 Hz), 7.79 (2H, d, *J* = 8.1 Hz), 3.89 (6H, q, *J* = 7.1 Hz), 2.63 (3H, s), 1.26 (9H, t, *J* = 7.1 Hz); ^13^C-NMR (CDCl_3_) δ 198.7, 138.6, 137.5, 135.3, 127.5, 59.1, 26.9, 18.4.

*1-(4-(Triethoxysilyl)phenyl)-4,4,4-trifluoro-1,3-butanedione* (**2**). The general approach described by Zayia [15] was followed with several modifications. A 50-mL round-bottom three-neck reaction flask was flame-dried under vacuum and was equipped with a magnetic stirring bar, three rubber septa and a positive-pressure N_2_ inlet line. The flask was charged with a solution of **7** (1.00 g, 3.55 mmol) dissolved in anhydrous diethyl ether (15 mL). The flask was cooled to -78^°^C, and with stirring, a 1.5 M solution of LDA in cyclohexane (2.61 mL, 3.91 mmol of LDA) was added dropwise with a syringe over the course of 2 min and the resulting mixture was stirred at -78^°^C for an additional 90 min. With continued cooling and stirring, neat 2,2,2-trifluoroethyl trifluoroacetate (2.38 mL, 3.48 g, 17.75 mmol) was quickly added over the course of 1 min with a syringe and the resulting mixture was stirred at -78^°^C for 4 h. Neat H_2_SO_4_ (0.40 mL, 0.732 g, 7.46 mmol) was then added in one portion with vigorous stirring using a microsyringe and the reaction mixture was allowed to warm to RT. The mixture was poured into a 125-mL separatory funnel containing ether (25 mL) and water (25 mL), the organic layer was isolated, and was washed with water, 10% aqueous NaHCO_3_ and saturated aqueous NaCl, dried (MgSO_4_) and concentrated *in vacuo* to afford the title diketone **2** as a thick, clear oil that was used without further purification: 0.98 g (2.59 mmol, 73%); FT IR (thin film) 3520-3280 (mod , O-H), 1605 (vs and broad, enolic form), and 1162 cm-1 (vs and broad, C-O); ^1^H-NMR (CDCl_3_) d 14.9 (1H, bs), 7.95 (2H, d, *J* = 7.8 Hz), 7.80 (2H, d, *J* = 7.8 Hz), 6.62 (1H, s), 3.87 (6H, q, *J* = 7.2 Hz), 1.31 (9H, t, *J* = 7.2 Hz); ^13^C-NMR (CDCl_3_) d 185.8, 177.8 (q, ^2^J_CF_ = 35.9 Hz), 138.9, 135.3, 130.3, 126.5, 117.1 (q, , ^1^J_CF_ = 284.3 Hz), 92.5, 59.0, 18.2. The product as isolated was found to be of sufficient purity for use in the subsequent sol-gel studies. Attempts to obtain an analytical sample of **2** *via* vacuum distillation failed due to the labile nature of the product though it was possible to obtain an enriched fraction of >95% purity (bp 142-146^°^C, Kugelrohr, 0.20 mmHg).
